# Differential utilization of surface and arboreal water bodies by birds and mammals in a seasonally dry Neotropical forest in southern Mexico

**DOI:** 10.1002/ece3.10781

**Published:** 2023-11-28

**Authors:** Carlos M. Delgado‐Martínez, Melanie Kolb, Fermín Pascual‐Ramírez, Eduardo Mendoza

**Affiliations:** ^1^ Posgrado en Ciencias Biológicas, Universidad Nacional Autónoma de México Unidad de Posgrado, Edificio D, 1er Piso Ciudad de México Coyoacán Mexico; ^2^ Instituto de Geografía, Universidad Nacional Autónoma de México Circuito exterior s/n, Ciudad Universitaria Ciudad de México Coyoacán Mexico; ^3^ Instituto de Investigaciones sobre los Recursos Naturales Universidad Michoacana de San Nicolás de Hidalgo Morelia Michoacán Mexico; ^4^ Instituto de Investigaciones en Ecosistemas y Sustentabilidad Universidad Nacional Autónoma de México Morelia Michoacán Mexico

**Keywords:** Calakmul, dendrotelmata, resource partitioning, Selva Maya

## Abstract

Water availability significantly influences bird and mammal ecology in terrestrial ecosystems. However, our understanding of the role of water as a limiting resource for birds and mammals remains partial because most of the studies have focused on surface water bodies in desert and semi‐desert ecosystems. This study assessed the use of two types of surface water bodies (waterholes and epikarst rock pools) and one arboreal (water‐filled tree holes) by birds and mammals in the seasonally dry tropical forests of the Calakmul Biosphere Reserve in southern Mexico. We deployed camera traps in 23 waterholes, 22 rock pools, and 19 water‐filled tree holes in this karstic region to record visits by small, medium, and large‐bodied birds and mammals during the dry and rainy seasons. These cameras were set up for recording videos documenting when animals were making use of water for drinking, bathing, or both. We compared the species diversity and composition of bird and mammal assemblages using the different types of water bodies by calculating Hill numbers and conducting nonmetric multidimensional scaling (NMDS), indicator species, and contingency table analyses. There was a greater species richness of birds and mammals using surface water bodies than tree holes during both seasons. There were significant differences in species composition among bird assemblages using the different water bodies, but dominant species and diversity remained the same. Terrestrial and larger mammalian species preferentially used surface water bodies, whereas arboreal and scansorial small and medium mammals were more common in arboreal water bodies. These findings suggest that differences in water body characteristics might favor segregation in mammal activity. The different water bodies may act as alternative water sources for birds and complementary sources for mammals, potentially favoring species coexistence and increasing community resilience to environmental variation (e.g., fluctuations in water availability). Understanding how differences in water bodies favor species coexistence and community resilience is of great relevance from a basic ecological perspective but is also crucial for anticipating the effects that the increased demand for water by humans and climate change can have on wildlife viability.

## Graphical abstract

This study examined the utilization of surface and arboreal water sources by birds and mammals in a seasonally dry tropical forest in southern Mexico. Camera traps were deployed in waterholes, rock pools, and water‐filled tree holes in the Calakmul Biosphere Reserve, Mexico. Bird and mammal species richness was higher in surface water bodies, with notable differences in usage patterns. Larger mammals preferred surface water, while arboreal mammals favored tree holes. The findings highlight the potential for water sources to influence species coexistence and community resilience in response to environmental changes.

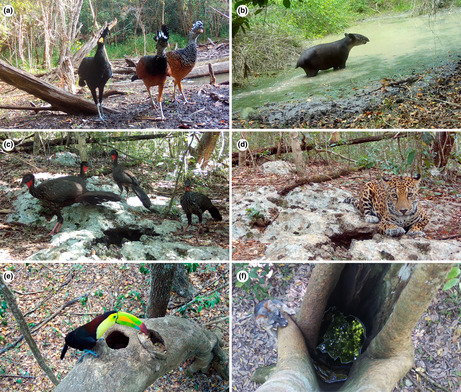

## 1 INTRODUCTION

The supply of essential resources strongly affects the distribution and abundance of animal species (Hamilton & Murphy, 2018; Messier, 1991). These effects can escalate to affect the structure and dynamics of animal communities. Examples of limiting resources affecting bird and mammal communities include shared prey (Gilg et al., 2003), nectar‐producing flowers (Guevara et al., 2023), and cavities for nesting birds (Jiménez‐Franco et al., 2018).

Water availability is a clear example of a limiting factor having a significant impact on the ecology of birds and mammals in most terrestrial ecosystems. Animals depend on water sources for drinking, bathing, cooling, and preying, among other things (Clayton et al., 2010; Gossner et al., 2020; Hafez, 1964; Lee et al., 2017). Variation in water availability is linked to fluctuations in mammal populations (Gandiwa et al., 2016), movement patterns (Chamaillé‐Jammes et al., 2016), and habitat connectivity (O'Farrill et al., 2014). Several studies have incorporated water body features as covariates to analyze ecological parameters such as mammal occupancy (e.g., Di Bitetti et al., 2020), but less research has focused directly on analyzing the characteristics of the use of water bodies by vertebrates. Furthermore, most of these studies are concentrated in arid regions (e.g., Amoroso et al., 2020; Edwards et al., 2016; Harris et al., 2015), limiting our understanding of the role of water as a limiting factor for vertebrates in other ecosystems.

Resource partitioning has been proposed as a mechanism for animal species to reduce antagonistic interactions (e.g., competition and predation) occurring when exploiting limited resources (Schoener, 1974; Walter, 1991). This adaptive mechanism allows interacting species to coexist by specializing in different resource sources or by utilizing them at different times or places (Lear et al., 2021).

Tropical birds and mammals have evolved different strategies to exploit water sources. They can use water in rivers and pools (e.g., Stommel et al., 2016) or tank epiphytes and water‐filled tree holes in the canopy (e.g., Sharma et al., 2016). Some species, such as scansorial mammals and birds, likely have more flexibility to use surface and canopy water sources, but for other animals, access might be more limited. The interplay between the variation in water source characteristics and types of animal locomotion opens the possibility for resource partitioning to occur. Previous studies have focused on analyzing the temporal partitioning of water use by mammals (Adams & Thibault, 2006; Edwards et al., 2017; Valeix et al., 2007), but until now, the dynamics of the use of surface and arboreal water bodies at the community level are unknown. A better knowledge of the strategies involved in the use of water sources by birds and mammals is essential to improve our understanding of species coexistence mechanisms and to predict the impacts of anthropogenic disturbances such as those associated with climate change (Galetti et al., 2016; Votto et al., 2020).

Seasonally dry tropical forests are water‐stressed ecosystems rich in vertebrate species, offering an ideal opportunity to study resource partitioning (Allen et al., 2017; Mooney et al., 1995; Ocón et al., 2021). Seasonal forests growing on karstic soils, such as those occurring in the Calakmul region in southern Mexico, undergo a particularly marked limitation in water availability due to the absence of permanent, extensive surface water bodies caused by fast water infiltration (García‐Gil et al., 2002). There is some evidence showing the significant impact variation in water availability has on wildlife distribution and survival in the Calakmul region. For instance, white‐lipped peccaries concentrate their activity around waterholes during the dry season, and a documented peak in tapir deaths coincided with a marked drought in the year 2019 (Reyna‐Hurtado et al., 2012, 2019). The congregation of species in waterholes might result in a greater risk of attacks, such as suggested by the documented kill of an ocelot by a jaguar in the nearby Guatemala Maya forest (Perera‐Romero et al., 2021). In Calakmul, wildlife can obtain water not only from waterholes but also from epikarst rock pools and water‐filled tree holes. Each of these water bodies has features that can attract different species (see full description in Section 2). Some studies have individually documented the extensive use of these water sources by birds and mammals, but a comparative approach is missing (Delgado‐Martínez, Alvarado, et al., 2022; Delgado‐Martínez, Cudney‐Valenzuela, & Mendoza, 2022; Reyna‐Hurtado et al., 2012).

The Calakmul region stands out globally due to its high biodiversity (Myers et al., 2000). However, it is currently facing escalating pressure due to anthropogenically‐driven factors such as deforestation and climate change (Mardero et al., 2020; Ramírez‐Delgado et al., 2014). This situation poses a significant risk to water quality and availability, which can have far‐reaching consequences for vertebrate populations. This research evaluates if there exists a partitioning in the use of water bodies by small, medium, and large‐bodied birds and mammals in the seasonally dry tropical forest occurring in the Calakmul region. Specifically, we assessed the species diversity and composition of bird and mammal assemblages using two types of surface water bodies (waterholes and epikarst rock pools) and one type of arboreal water body (water‐filled tree holes). Moreover, we analyzed how differences in water source use between birds and mammals are related to contrasts in functional traits such as locomotion and body size. We hypothesize that there will be distinct patterns of water source use between birds and mammals and that differences among species within these groups will be associated with variation in their functional traits. We expect to find more pronounced differences in water use among species within animal groups during the rainy season, when water is more widely available and, therefore, animals have a greater opportunity to select.

## 2 METHODS

### 2.1 Study area

**FIGURE 1:**
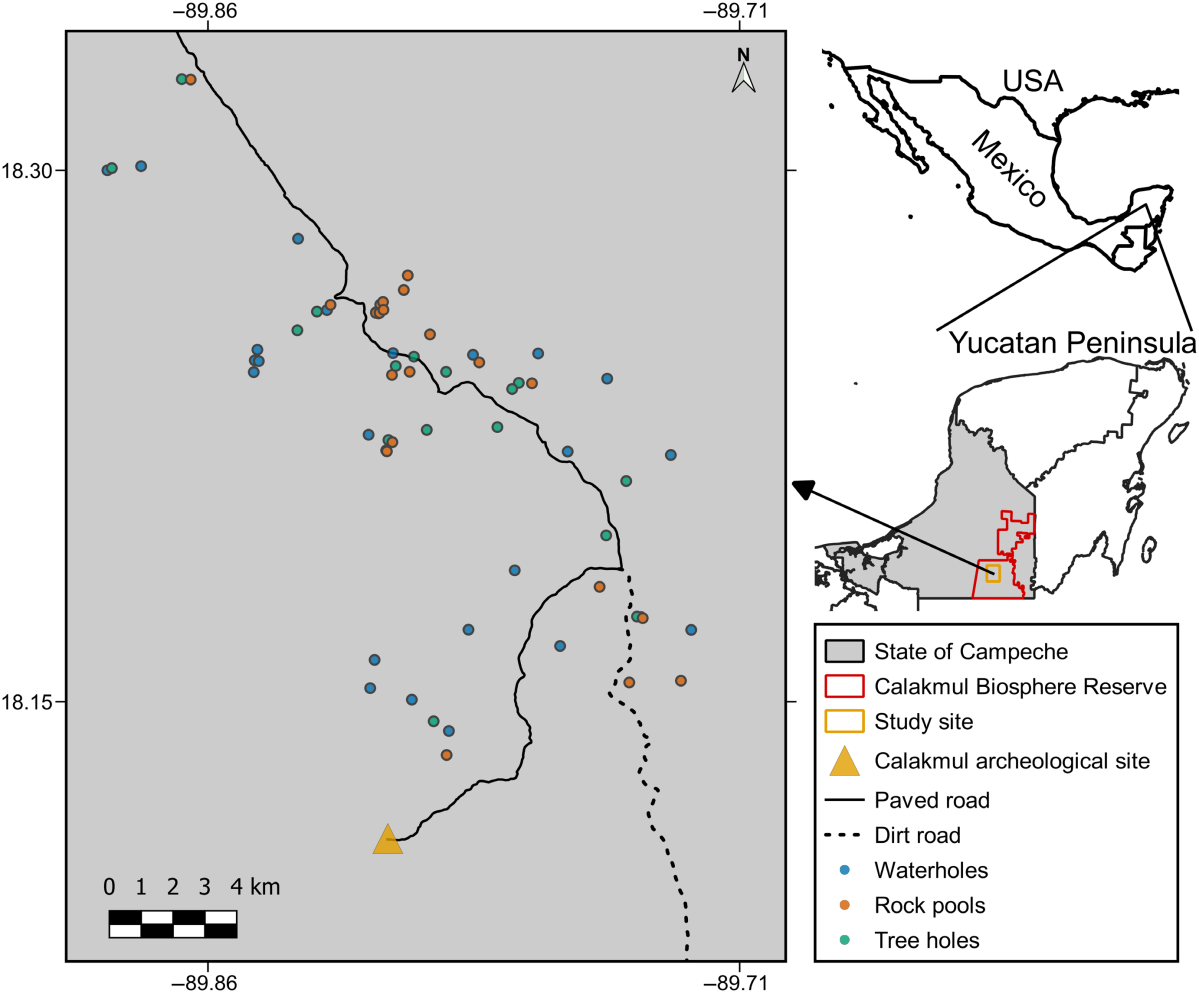
Study area and distribution of sampled water bodies in the Calakmul Biosphere Reserve, Campeche, Mexico.

Fieldwork was carried out in the southern portion of the buffer zone of the Calakmul Biosphere Reserve (CBR, 89°43′26″–89°49′23″ W, 18°16′01″–18°8′49″ N), in the state of Campeche, southern Mexico (Figure 1). The CBR was created in 1989 and has an extent of 723,185 ha, constituting the largest tropical protected area in Mexico (Galindo‐Leal, 1999; Gómez‐Pompa & Dirzo, 1995). The study area has an approximate extent of 63,000 ha and supports continuous vegetation, except for a narrow road (ca. 4 m wide) leading to the Calakmul Archeological site. During this study, only ecotourism and research were allowed in the study area, and no primary activities have occurred there since the establishment of the CBR (INE, 1999). The CBR is critically important for the conservation of biodiversity in Latin America due to the fact that it supports some of the largest populations of iconic species of Mesoamerica, such as *Tapirus bairdii* and *Panthera onca* (Ceballos et al., 2021; Naranjo, 2018). Together with the Maya Biosphere Reserve in Guatemala, the CBR constitutes the largest tract of tropical forest in Mesoamerica (Potapov et al., 2017). However, like most of the tropics, this region is under increasing human pressure due to activities such as illegal selective logging, hunting, deforestation for farming and cattle ranching, and more recently, infrastructure development (Ramírez‐Delgado et al., 2014; Špirić et al., 2022).

The CBR has a tropical wet and dry climate with a dry winter (Köppen‐Geiger classification: Aw; Beck et al., 2018). The region has a marked precipitation seasonality with a rainy season occurring from May to October and a dry season from November to April (monthly precipitation is <60 mm) (Mardero et al., 2020; Vidal‐Zepeda, 2005) with a mean annual precipitation of 1076 mm (CONAGUA, 2023; Martínez & Galindo‐Leal, 2002). The average annual temperature is 25.7°C, whereas the average annual minimum and maximum temperature are 18.7 and 32.8°C, respectively (CONAGUA, 2023; Figure S1). According to the standardized precipitation index calculated for the region, the amount of precipitation during our study period was within the range of normal variation (CONAGUA, 2023).

#### 2.1.1 Geology and hydrology in the CBR

**FIGURE 2:**
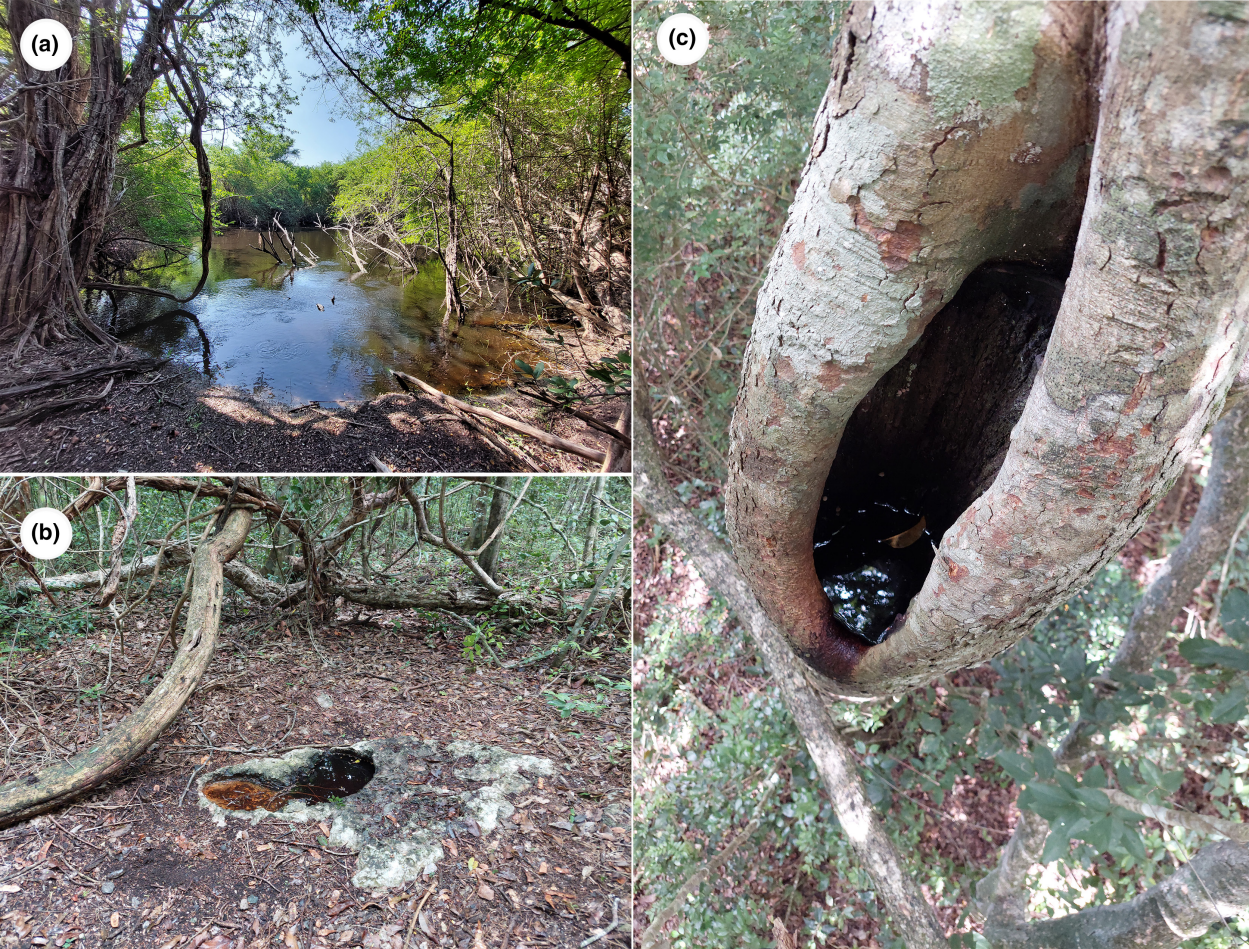
Target water bodies in the Calakmul Biosphere Reserve, Campeche, Mexico: (a) waterhole, (b) rock pool, and (c) water‐filled tree hole.

The CBR is located on the karstic landscape of the Petén Plateau which is primarily composed of limestone, shaped by chemical weathering and erosion (Ensley et al., 2021; Torrescano‐Valle & Folan, 2015). Water bodies within the study site depend on precipitation as their main source of water due to the absence of perennial streams. The following are the most common water bodies in the study site and are the focus of this study. Waterholes. These water bodies, locally known as *aguadas*, are dolines resulting from the dissolution of limestone that, together with clay accumulation, reduces water percolation and promotes the accumulation of rainfall (Figure 2a; Back & Lesser, 1981; García‐Gil et al., 2002; Kranjc, 2013). Waterholes have an approximate density of one per 10.5 km^2^ in the CBR and vary in size from 10 to 40,000 m^2^ but typically they do not exceed 5000 m^2^ (Reyna‐Hurtado et al., 2012). Usually, a high proportion of the waterholes dries up during the dry season, remaining only the largest. During extreme drought events, even the largest waterholes can be gone (O’Farrill et al., 2014).

Epikarst rock pools. These water bodies, locally known as *sartenejas*, are classified as *kamenitzas* in geological terms and are natural depressions in land formed by the dissolution of exposed bedrock (Lundberg, 2013). Rock pools usually occur on elevated ground or hilltops in the study area (Figure 2b; Flores, 1983). There is approximately one rock pool per 0.1 km^2^, usually covering less than a square meter (C. M. Delgado‐Martínez, unpublished data). Rock pools collect water even during moderate precipitation events and can store it for several weeks (C. M. Delgado‐Martínez, personal observation; Reyna‐Hurtado et al., 2012).

Water‐filled tree holes. These water bodies are formed in cavities or depressions in trees, where rainwater accumulates (Figure 2c; Kitching, 2000). The density and average volume of water‐filled tree holes in the Calakmul region are unknown. As rock pools, most tree holes have ephemeral hydroperiods, but some of them can retain water during the whole year (C. M. Delgado‐Martínez, personal observation).

### 2.2 Searching and sampling of water bodies

We compiled information on the location of 24 waterholes, 37 rock pools, and 73 tree holes based on existing information for waterholes (García‐Gil et al., 2002), previous studies (Delgado‐Martínez, Alvarado, et al., 2022; Delgado‐Martínez, Cudney‐Valenzuela, & Mendoza, 2022), and fieldwork conducted for this study (ca. 150 km of search by foot). From these water bodies, we selected 23 waterholes, 22 rock pools, and 19 tree holes. The selection of waterholes was based on whether they had water during our initial visit, which occurred in July–August 2021 (these months corresponded to the start of the rainy season). On the other hand, we only selected rock pools with an estimated volume >10 L. For the selection of tree holes, we applied the following criteria: (1) to have their entrance at least 1 m above the ground; (2) to have a minor axis of the entrance >10 cm; and (3) to have a depth >20 cm. These criteria were aimed at increasing the probability of the different water bodies maintaining water throughout the study period. To prevent spatial autocorrelation, we avoided monitoring water bodies of the same type, <500 m apart, at the same time.

Due to logistic limitations, we were not able to sample all the water bodies simultaneously. Therefore, we divided them into three groups, including 7–8 water bodies of each type. These groups were monitored sequentially, with one camera trap aimed at each water body for at least 45 days. After the completion of this period, camera traps were moved to the next group of water bodies. We repeated this procedure to complete one rainy season and one dry season for each group of water bodies (from July 2021 to September 2022). Camera traps in the waterholes and rock pools were installed at heights ranging from 40 to 70 cm and at distances of 2–4 m from the water edges. We used trail camera holders (HME‐TCH‐SO) to set the camera traps focused on the tree holes. In most cases, these holders were fastened to the same tree where the tree hole was located, approximately 2 m from the entrance to the tree hole, to secure a direct view of its entrance. When it was not possible to fasten the camera holder to the same tree where the tree hole was located, we fastened it to a nearby tree. A single camera was enough to fully monitor the entire rock pools and tree holes, but not the waterholes. To reduce the probability of missing some animal species visiting waterholes, we followed two strategies: (a) we aimed the cameras preferentially at areas with evidence of animal activity, such as footprints or peccary wallows, and (b) we made sure to have a clear view of the areas within the waterholes that maintain water even in the dry season. We used camera trap models Browning Spec Ops Elite HP4, Browning Strike Force Elite BTC5HDE, and Bushnell Trophy Cam HD Aggressor 119876C programmed to take 20‐s long videos each time they were activated and to have a 5‐s delay before reactivation.

### 2.3 Data processing

We tagged videos with the identity of the species recorded and made a database adding information about their locomotion (i.e., arboreal, scansorial, and terrestrial) and body mass (González‐Salazar, 2013). We classified the species into three categories based on their body mass: small, medium, and large. Birds with a body mass lower than 0.5 kg were classified as small, those with a body mass higher than 0.5 kg but lower than 2 kg as medium, and birds with a body mass higher than 2 kg as large. Mammals with a body mass lower than 4 kg were classified as small, those with a body mass higher than 4 kg but lower than 13 kg as medium, and mammals with a body mass higher than 13 kg as large. The categories were defined to have an approximately equal number of species in each group.

To reduce sources of bias during the statistical analysis, we discarded records of aquatic birds and species with a body mass <220 g (which can be missed by camera traps more frequently) or found in less than four sites. We grouped videos of the same species recorded consecutively and with the same camera, following the methodology described in Camargo‐Sanabria and Mendoza (2016). These grouped videos were classified as visitations. We calculated the frequency of visitation for each animal species using the following equation: (number of visits/sampling effort) × 100 camera trap days (O'Brien et al., 2003). The sampling effort was equal to the total number of days a camera trap was active. To ensure recorded visits used for analyses were primarily driven by animals' attraction to water, we only considered those in which it was clear they were drinking, bathing, or both. During the review of videos, we identified some common behaviors, such as scent‐marking in the case of carnivores, digging into the organic material found in rock pools and tree holes, grooming among peccaries, and even frog and turtle hunting by ocelots.

### 2.4 Data analysis

#### 2.4.1 Comparison of bird and mammal species richness and diversity among water bodies

We used a sample coverage analysis (Chao & Jost, 2012) to estimate the completeness of bird and mammal surveys in each type of water body (i.e., waterholes, rock pools, and tree holes). For each type of water body, we generated sample‐based rarefaction and extrapolation curves of Hill numbers (*q* = 0, 1, and 2) with their corresponding 95% confidence intervals (Chao et al., 2014). We compared the curves of the different types of water sources and their corresponding 95% confidence intervals within the same season. These analyses were performed using the *iNEXT* R package (Hsieh et al., 2022).

#### 2.4.2 Comparison of bird and mammal species composition among water bodies

We conducted a non‐metric multidimensional scaling (NMDS) analysis to test for differences in the species composition of groups of birds and mammals visiting each type of water body. We calculated the Bray‐Curtis index, based on the species' frequencies of visitation, to use it as a measure of distance in the NMDS. To test for the statistical significance of species clusters indicated by the NMDS, we analyzed similarities (ANOSIM) using 10,000 permutations. We used the *vegan* R package to conduct these analyses (Oksanen et al., 2022).

To assess if there were animal species associated with specific water bodies, we conducted an indicator species analysis using 10,000 permutations with the R package *indicspecies* (De Caceres & Legendre, 2009). By comparing observed data to randomized permutations, this analysis determines which species have a nonrandom association with a particular type of water body. Finally, to assess if capture frequencies of the different locomotion types and body mass categories differed among the species visiting the three water bodies, we aggregated the frequencies and conducted a contingency table analysis (Franke et al., 2012). In cases where the resulting *p*‐value was below .05, we calculated the adjusted residuals to measure the standardized differences between the observed and expected frequencies.

## 3 RESULTS

**FIGURE 3:**
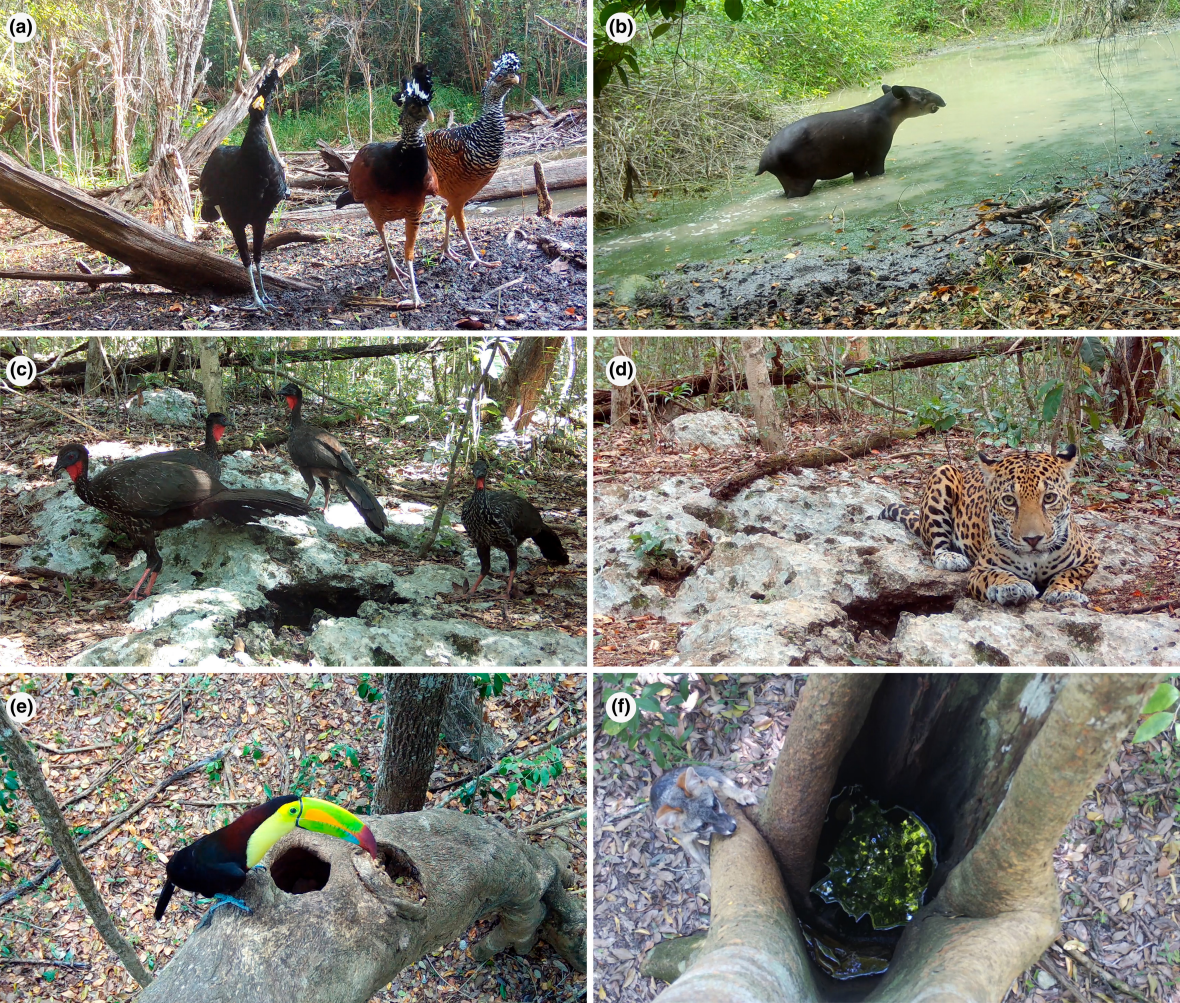
A sample of the birds and mammals using different water bodies in the Calakmul Biosphere Reserve. (a) *Crax rubra* and (b) *Tapirus bairdii* recorded at waterholes; (c) *Penelope purpurascens* and (d) *Panthera onca* recorded at a rockpool; (e) *Ramphastos sulfuratus* and (f) *Urocyon cinereoargenteus* observed in water‐filled tree holes.

We totalized a sampling effort of 6713 camera trap days, with 2203 days in the waterholes, 2828 days in the rock pools, and 1682 days in the tree holes (Table S1). We recorded 43 species in total (18 birds and 25 mammals; Figure 3 and Table S2) visiting the three water sources: 15 bird and 20 mammal species in the waterholes, 8 bird and 11 mammal species in the tree holes, and 13 bird and 19 mammal species in the rock pools. *Crax rubra* was the most observed bird species throughout both the dry and rainy seasons in the three types of water bodies. During the dry season, *Philander opossum* was the most common mammal species in the waterholes, while *Sciurus deppei* was the most common in the tree holes and *Pecari tajacu* in the rock pools. In contrast, during the rainy season, *Odocoileus virginianus* was the most common mammal species in the waterholes, followed by *Urocyon cinereoargenteus* in the tree holes, and *Cuniculus paca* in the rock pools (Figure S2).

### 3.1 Differences in animal species richness and diversity among water bodies

**FIGURE 4:**
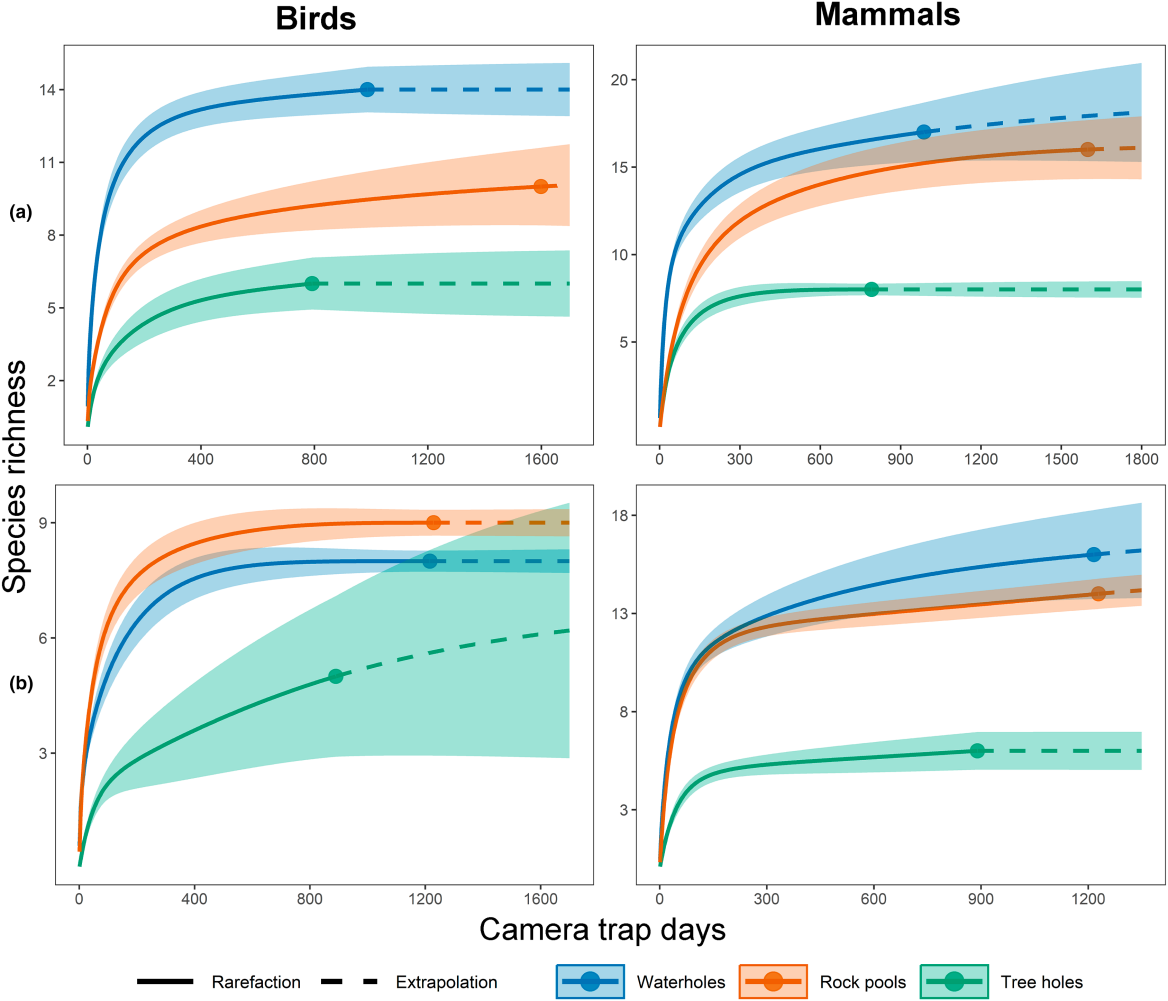
Species richness of birds and mammals visiting the waterholes, tree holes, and rock pools during the (a) dry and (b) rainy seasons in the Calakmul Biosphere Reserve, Campeche, Mexico.

The sample coverage of bird species in the different water bodies was very high (above 99%) during both the dry and rainy seasons (Table S3); only the tree holes had a slightly lower coverage in the rainy season (95.2%). During the dry season, bird species richness (q0) was highest in the waterholes and lowest in the tree holes (Figure 4). Likewise, Shannon diversity (q1) was highest in the waterholes, while the tree holes and rock pools had similar levels of diversity (Figure S3). Finally, Simpson diversity (q2) was higher in the waterholes than in the rock pools but indistinguishable from tree holes (Figure S4). In comparison, in the rainy season, bird species richness (q0) was higher in the rock pools than in the waterholes, whereas tree holes were indistinguishable from the other water bodies (i.e., their 95% confidence intervals overlapped). Similarly, the confidence intervals of the tree hole curve overlapped with the curves of other water bodies when examining Shannon and Simpson diversity.

Sample coverage of mammal species was also very high (above 99%) during both seasons (Table S4). In the dry season, mammal species richness (q0) and Shannon diversity (q1) were similar in the waterholes and rock pools but lower in the tree holes (Figure 4; Figure S3). Simpson diversity (q2) was higher in the rock pools and lower in the waterholes; diversity in these two water sources was indistinguishable from that found in tree holes (Figure S4). In the rainy season, species richness (q0) and Shannon (q1) diversity had a similar pattern, with waterholes and rock pools having greater diversity and tree holes lower. Simpson diversity was lowest in tree holes and highest in the rock pools.

### 3.2 Differences in bird and mammal species composition

**FIGURE 5:**
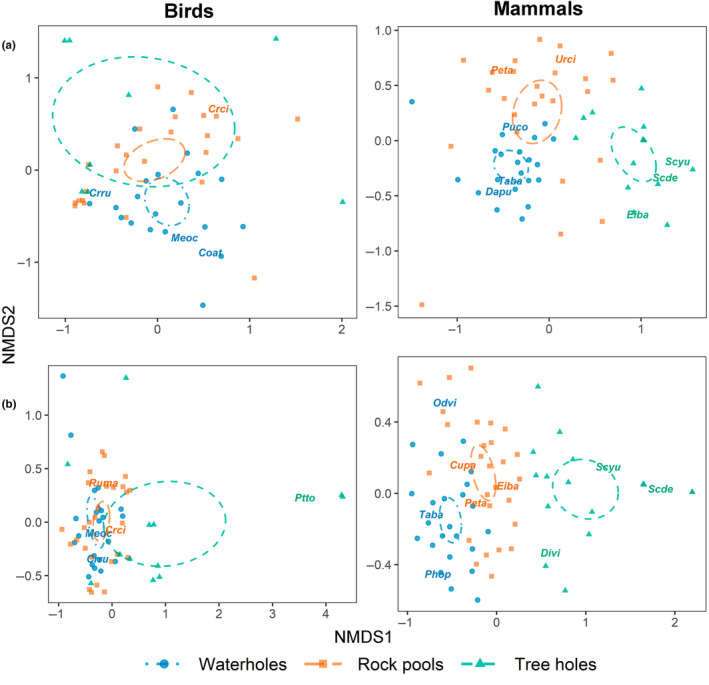
Species composition of birds and mammals that visited the target water bodies during the (a) dry and (b) rainy seasons. Bird species codes: *Coat*, *Coragyps atratus*; *Crru*, *Crax rubra*; *Crci*, *Crypturellus cinnamomeus*; *Meoc*, *Meleagris ocellata*; *Ptto*, *Pteroglossus torquatus*; and *Ruma*, *Rupornis magnirostris*. Mammal species codes: *Cupa*, *Cuniculus paca*; *Dapu*, *Dasyprocta punctata*; *Divi*, *Didelphis virginiana*; *Eiba*, *Eira barbara*; *Odvi*, *Odocoileus virginianus*; *Peta*, *Pecari tajacu*; *Phop*, *Philander opossum*; *Puco*, *Puma concolor*; *Scde*, *Sciurus deppei*; *Scyu*, *Sciurus yucatanensis*; *Taba*, *Tapirus bairdii*; *Urci*, *Urocyon cinereoargenteus*.

During both the dry (ANOSIM; *R* = .22, *p* < .001) and rainy seasons (*R* = .28, *p* < .001), the bird assemblages visiting the different water sources displayed a moderate yet statistically significant dissimilarity. In the dry season, *C. rubra*, *Coragyps atratus*, and *Meleagris ocellata* were associated with waterholes, while *Crypturellus cinnamomeus* was associated with rock pools (Figure 5). In the rainy season, *C. rubra* and *M. ocellata* were associated with waterholes, whereas *Pteroglossus torquatus* was associated with tree holes, and both *C. cinnamomeus* and *Rupornis magnirostris* were associated with rock pools.

We identified a significant association between the locomotion categories of birds and their occurrence in the different water body types (Figure S5). In the dry season (*χ* ^2^ = 1113.2, df = 4, *p* < .001), terrestrial birds exhibited a higher frequency in waterholes, while scansorial species visited them less frequently. Moreover, arboreal and scansorial birds showed a greater frequency in the tree holes, whereas terrestrial species displayed a lower visitation rate. Interestingly, rock pools received fewer visits from both arboreal and terrestrial birds. In contrast, during the rainy season (*χ* ^2^ = 2514.4, df = 4, *p* < .001), scansorial birds had a higher frequency of visits to waterholes, while arboreal species visited them less frequently. Additionally, arboreal birds exhibited a higher preference for tree holes, while scansorial and terrestrial species showed lower visitation rates. There was not a bird group showing a specific association with rock pools.

We also observed a relationship between the body mass categories of birds and their occurrence in the different water body types (Figure S6). In the dry season (*χ* ^2^ = 836.96, df = 4, *p* < .001), medium‐sized birds were more frequently recorded in tree holes, while small and large species showed lower frequencies. Rock pools received more visits from small birds and fewer from medium species. Only waterholes were not visited by any bird group at a frequency greater than expected by chance. In the rainy season (*χ* ^2^ = 1310.5, df = 4, *p* < .001), waterholes were frequented more by large birds and less by small and medium‐sized birds. Both tree holes and rock pools were visited more by small and medium‐sized birds and less by large species.

Differences in mammal composition among the water bodies were more pronounced than those observed among bird assemblages, both during the dry season (ANOSIM; *R* = .43, *p* < .001) and the rainy season (*R* = .50, *p* < .001). During the dry season, *Dasyprocta punctata*, *Puma concolor*, and *T. bairdii* were associated with waterholes, while *Sciurus* spp. and *Eira barbara* were associated with tree holes, and *P. tajacu* and *U. cinereoargenteus* with rock pools (Figure 5). In the rainy season, *O. virginianus*, *P. opossum*, and *T. bairdii* were associated with waterholes; *Didelphis virginiana* and *Sciurus* spp. with tree holes and *C. paca*; *E. barbara*, and *P. tajacu* with rock pools.

We found a significant association between the locomotion categories of mammals and their occurrence in the different water body types (Figure S5). In the dry season (*χ* ^2^ = 10,378, df = 4, *p* < .001), waterholes were more frequented by scansorial and terrestrial mammals, while arboreal mammals visited them less frequently. In the rainy season (*χ* ^2^ = 5901.7, df = 4, *p* < .001), waterholes were primarily visited by terrestrial mammals. Across both seasons, tree holes were preferred by arboreal and scansorial mammals, while terrestrial species showed lower visitation rates. Similarly, rock pools attracted more terrestrial mammals and were less frequently visited by arboreal and scansorial species, regardless of the season.

Likewise, we found significant associations between the body mass of mammals and water body types (Figure S6). During the dry season (*χ* ^2^ = 5373.7, df = 4, *p* < .001), waterholes had a higher frequency of visits by large mammals and a lower frequency by small and medium species. Tree holes were more frequently visited by small and medium mammals, while large mammals showed a reduced tendency to visit them. Rock pools had higher visitation by medium mammals and lower visitation by small species. In the rainy season (*χ* ^2^ = 4296.3, df = 4, *p* < .001), waterholes were frequented more by small and large mammals, while medium species had a lower visitation rate. Tree holes were more frequently visited by small and medium mammals and less frequently by large mammals. Rock pools were more frequently visited by medium mammals and less frequently by small and large mammals.

## 4 DISCUSSION

This study shows novel information about the utilization of surface and arboreal water bodies by a diverse community of birds and mammals in a seasonally dry tropical forest. Our sampling effort made it possible to record a diverse community of birds and mammals in both seasons, showing that the three types of water bodies are centers of high wildlife activity. Notably, our records documented the presence of elusive species of significant conservation interest, including *T. pecari* and *Mazama pandora* in both waterholes and rock pools.

Our findings provided support for our initial hypothesis, showing contrasting responses between birds and mammals, with more pronounced differences in water partitioning among species in the latter group. We consistently observed higher species richness and diversity of mammals in surface water bodies, with larger species concentrating their activity in the waterholes and arboreal and scansorial species in the tree holes; albeit infrequently, we recorded large species, such as *P. onca*, *Leopardus pardalis*, and *U. cinereoargenteus*, climbing to use tree holes. These differences were more marked during the rainy season, as we predicted.

In ecosystems with limited water sources, species often congregate around the few available water bodies, particularly during the dry season, leading to potential negative interactions with dominant species (Ferry et al., 2016). Nevertheless, when multiple water sources are available in different locations with varying accessibility, such as ground versus canopy, resource partitioning can occur, enabling differential resource use. Resource partitioning is widely recognized as a key mechanism for promoting species coexistence and maintaining biodiversity by reducing interspecific competition (Chesson, 2000; Schoener, 1974). This mechanism has been documented in tropical bird and mammal species, including vertical stratification (e.g., Akkawi et al., 2020; Ferreguetti et al., 2018; Mohd‐Azlan et al., 2014; Sushma & Singh, 2006). Both waterholes and rock pools exhibited similar mammal species richness and diversity in both the dry and rainy seasons, which were greater than for the tree holes. The presence of comparable diversity in these surface water sources highlights their importance in supporting a diverse mammal community throughout the year (Delgado‐Martínez, Alvarado, et al., 2022; Reyna‐Hurtado et al., 2010). Yet, our analyses also showed the existence of differences in species composition of the animal assemblages visiting the three water body types. These findings suggest that the analyzed water bodies have ecological features that increase their probability of being visited by specific mammalian species. Thus, for the mammal community, the water bodies may be acting as complementary sources (Mallinger et al., 2016; Maurer et al., 2022). This could potentially enable a greater number of mammal species to coexist within this water‐stressed ecosystem (Martins et al., 2018; Thomsen et al., 2022), as resources in a community are considered complementary when different species use them in a way that avoids direct competition (Cleland, 2011; Tilman, 1982).

In contrast, in the case of bird assemblages, we found that a substantial proportion of species were shared and the identity of the dominant species was maintained, despite there being significant differences in species composition. Although we found an association between predominantly terrestrial bird species (e.g., *M. ocellata*) and surface water bodies, none of the recorded bird species is completely flightless (Greenwood, 2001). Their ability to utilize the canopy provides them with greater mobility and potential access to alternative resources (Partasasmita et al., 2017; Winkler & Preleuthner, 2001). Therefore, the relatively less marked differential water use by birds can be attributed to their capacity to exploit vertical space, reducing their dependence on specific water sources. Consequently, these results suggest that the different water bodies may be acting, to some degree, as alternative water sources for birds (Tilman, 1982), leading to similarities in the species composition of the assemblages recorded in each of them.

Alternatively, the moderate dissimilarities in bird species composition observed across different water bodies could be explained by the fact that species in this group may adopt distinct temporal patterns of use of this resource as a strategy to minimize direct competition and predation risk (Harmange et al., 2021; Olea et al., 2022). However, no studies have focused on the temporal partitioning of water sources by birds. Such temporal segregation could potentially allow birds to access water sources without engaging in antagonistic interactions, but this possibility warrants further research.

An additional factor influencing the differential use of water sources can be the perceived predation risk associated with the visitation to each type of water body (Periquet et al., 2010; Valeix et al., 2009). Large terrestrial predator species, such as *P*. *onca* and *P. concolor*, were associated with waterholes during the dry season and with waterholes and rock pools during the rainy season. The presence of these predators can contribute to the creation of a landscape of fear due to the potential for lethal interactions with prey species (Bleicher, 2017; Laundre et al., 2010; Perera‐Romero et al., 2021). To mitigate this risk, prey species may actively seek out alternative water sources, such as tree holes, where the probability of encounters with predators is lower (Doody et al., 2007; Hall et al., 2013). This could be the case for *P. tajacu* and *N. narica*, two of their main prey species (Núñez et al., 2000), which were more commonly recorded in rock pools and tree holes, respectively, during the dry season. Thus, predator avoidance behavior might interact with resource partitioning to contribute to the observed differential pattern of use of water sources.

The absence of detailed information regarding the presence or absence of water in rock pools and tree holes could potentially lead to an underestimation of visitation frequencies for some species in these water bodies. For example, in some cases, we were unable to confirm whether an animal had indeed drunk water or had merely inspected the site. Nevertheless, these cases were not included in the analyses, and we think that this probability of underestimation was consistent across different water bodies.

Although our study primarily focused on the type of water source as the main explanatory variable, it is important to acknowledge that several other factors can influence the selection and utilization of water sources by birds and mammals. Some potential variables, which were not included in our study but are known to impact the use of water bodies, encompass the characteristics of the vegetation growing within water bodies and their surrounding areas (Eakin et al., 2018; Votto et al., 2022), water body and landscape features such as water depth, terrain slope, distance to roads, and proximity to other water sources (Najafi et al., 2019; Pin et al., 2018), and food availability within and around the water bodies (Chaves et al., 2021; Eakin et al., 2018). Furthermore, in the case of tree holes, variations in height and connectivity of tree holes (e.g., through lianas and neighboring trees) may influence the utilization of these water sources (Cudney‐Valenzuela et al., 2021). Exploring the role of these factors would likely help to gain a more comprehensive understanding of water source selection by wildlife.

### 4.1 Implications for conservation

The Calakmul region, like other parts of the world, is experiencing an increase in drought frequency and disruptions in rainfall patterns due to global climate change, resulting in more uneven distribution of rainfall throughout the year and more intense rainfall events (Mardero et al., 2020). These changes have the potential to affect water availability for wildlife by altering the hydrological cycles of water bodies. Waterholes seem to be experiencing longer periods of drying, which can make them less available as water sources in the future (Reyna‐Hurtado et al., 2019). In contrast, tree holes and rock pools seem to be able to fill even with moderate rainfalls (C. M. Delgado‐Martínez, unpublished data). Therefore, species strongly associated with waterholes, such as *T*. *bairdii*, seem to be more prone to be affected by changes in rainfall patterns. Furthermore, a recent study in the region has documented that in times of scarcity, wildlife seeks water in human‐made places, such as cattle troughs and apiaries, which sometimes leads to human‐wildlife conflicts (Pérez‐Flores et al., 2021). This situation may become more common soon due to changes in water availability in the region.

Moreover, the Calakmul region is facing greater pressure due to increasing infrastructure development and human activity. In particular, the construction of the Maya Train, one of the most important infrastructure projects of the current federal administration, has the potential to impact regional biodiversity. The project aims to boost transportation and tourism, potentially benefiting local communities but increasing resource demands, particularly water (Camargo & Vázquez‐Maguirre, 2021; García et al., 2022). Some waterholes are a common attraction in the reserve; thus, an uncontrolled rise in tourist activity at waterholes has the potential to disrupt the behavior of sensitive species, causing their displacement to lower‐quality sites and potentially intensifying interspecific competition and predation risk (Crosmary et al., 2012; Zukerman et al., 2021). Additionally, the selective logging and clear‐cutting practices prevalent in the region can further reduce the availability of tree holes (Armenta‐Montero et al., 2020; Blakely & Didham, 2008), which are essential for arboreal and scansorial species. The scarcity of suitable tree holes may force these species to rely on surface water sources, consequently increasing their vulnerability to terrestrial predators. Therefore, overall, the combined impacts of human activities and climate change seem to have a great potential to affect the spatio‐temporal water distribution patterns, altering the patterns of water use by birds and mammals, which in turn might generate increased competition among species, greater predation risk, and potential human–wildlife conflicts.

Seasonally dry tropical forests are extensively distributed throughout the tropics and face similar threats to those affecting the Calakmul Biosphere Reserve (Siyum, 2020). However, significant knowledge gaps still exist concerning the ecology of these ecosystems and the effects of human activity on their functioning (Allen et al., 2017). Our findings help to increase our understanding of the role water source characteristics have on wildlife ecology and shed some light on the potential impacts anthropogenic activity can bring about on the fauna by the alterations of the patterns of water availability.

## AUTHOR CONTRIBUTIONS

**Carlos M. Delgado‐Martínez:** Conceptualization (lead); data curation (lead); formal analysis (lead); funding acquisition (lead); investigation (lead); methodology (lead); project administration (equal); software (lead); visualization (lead); writing – original draft (lead); writing – review and editing (equal). **Melanie Kolb:** Conceptualization (supporting); methodology (supporting); project administration (equal); supervision (equal); validation (equal); writing – review and editing (equal). **Fermín Pascual‐Ramírez:** Conceptualization (supporting); methodology (supporting); supervision (equal); validation (equal); writing – review and editing (equal). **Eduardo Mendoza:** Conceptualization (supporting); funding acquisition (supporting); methodology (supporting); project administration (equal); supervision (equal); validation (equal); writing – review and editing (equal).

## CONFLICT OF INTEREST STATEMENT

The authors declare no conflict of interest.

## Supporting information


Appendix S1.

## Data Availability

The data supporting the findings of this study, along with the code to conduct the analyses, are openly available in the Dryad Digital Repository: https://doi.org/10.5061/dryad.1jwstqk22.
