# Supplementary material for: Differential utilization of surface and arboreal water bodies by birds and mammals in a seasonally dry Neotropical forest in southern Mexico
Source: Ecol Evol. 2023 Nov 28;13(11):e10781. doi: 10.1002/ece3.10781 (PMC10682877; doi:10.1002/ece3.10781)
Supplement: Supplementary file 1 — Appendix S1. [file ECE3-13-e10781-s001.pdf]

Supplementary material for

Differential utilization of surface and arboreal water bodies by birds and mammals in a seasonally dry Neotropical forest in Southern Mexico

Carlos M. Delgado-Martínez, Melanie Kolb, Fermín Pascual-Ramírez and Eduardo Mendoza

Table S1. Accumulated sampling effort in the different types of water bodies.

	**Dry season**	**Rainy season**
Waterholes	987	1216
Rock pools	1599	1229
Tree holes	792	890

Table S2. Species recorded visiting the different water bodies. The quantities indicate the number of events.

	Waterholes		Rock pools		Tree holes	
	Dry	Rainy	Dry	Rainy	Dry	Rainy
**AVES**						
Buteogallus urubitinga	28	5	10	12	—	—
Cathartes aura	25	6*	—	—	—	—
Chondrohierax uncinatus	—	—	1*	—	—	—
Ciccaba virgata	51	9	28	22	3	2
Coragyps atratus	41	33*	—	—	—	—
Crax rubra	520	530	382	369	45	23
Crypturellus cinnamomeus	1	—	11	10	—	—
Ictinia plumbea	—	1*	—	—	—	—
Meleagris ocellata	176	124	20	25	—	—
Micrastur semitorquatus	4	4	5	4	—	1
Ortalis vetula	8	9	—	3	18	1
Penelope purpurascens	30	27	34	33	6	15
Psarocolius montezuma	—	—	2*	—	—	—
Pteroglossus torquatus	—	—	—	—	3*	5
Ramphastos sulfuratus	11	—	2	1*	4	1*
Rupornis magnirostris	14	7	34	55	—	—
Sarcoramphus papa	5*	1*	—	—	1*	—
Spizaetus ornatus	9	—	1	3*	—	—

Table S2. Continued.

	Waterholes		Rock pools		Tree holes	
	Dry	Rainy	Dry	Rainy	Dry	Rainy
**MAMMALIA**						
Bassariscus sumichrasti	—	—	—	—	—	2*
Caluromys derbianus	—	—	—	—	1*	—
Cuniculus paca	2	6	16	91	—	—
Dasyprocta punctata	58	84	4	41	—	—
Didelphis marsupialis	—	—	10	3*	—	—
Didelphis virginiana	7	2	12	19	21	24
Eira barbara	1	1	2	10	5	1
Herpailurus yagouaroundi	1*	1*	—	—	—	—
Leopardus pardalis	28	38	20	19	7	—
Leopardus wiedii	1*	—	4*	2*	2*	1*
Mazama pandora	5	3	1	13	—	—
Mazama temama	—	—	3*	—	—	—
Nasua narica	43	17	39	40	21	15
Odocoileus virginianus	54	186	15	55	—	—
Panthera onca	27	23	8	10	3	—
Pecari tajacu	7	28	49	62	—	—
Philander opossum	214	34	—	—	—	—
Procyon lotor	1*	1*	—	—	—	—
Puma concolor	57	27	—	—	14	16

Table S2. Continued.

	Waterholes		Rock pools		Tree holes	
	Dry	Rainy	Dry	Rainy	Dry	Rainy
Sciurus deppei	1	—	3	1	44	17
Sciurus yucatanensis	6	1	—	—	10	8
Spilogale angustifrons	—	—	2*	2*	—	—
Tapirus bairdii	75	62	4	1	—	—
Tayassu pecari	46	45	2	—	—	—
Urocyon cinereoargenteus	6	3	32	24	9	28

* These cases were excluded from the analyses because they were recorded only in three or fewer sites.

Table S3. **S**ummary of surveys of birds during the dry and rainy seasons. Q1 to Q10 shows the first ten incidence frequency counts. Q*i* is the number of species represented by exactly *i* records.

Water body	Observed species richness	Sample coverage	Q1	Q2	Q3	Q4	Q5	Q6	Q7	Q8	Q9	Q10
Dry season												
Waterholes	14	1	1	0	0	1	1	0	0	1	1	0
Rock pools	10	0.9981	1	1	0	0	1	0	0	0	0	1
Tree holes	6	1	1	0	1	1	0	1	0	0	0	0
Rainy season												
Waterholes	8	1	0	0	0	1	1	0	1	0	2	0
Rock pools	9	1	0	0	1	1	0	0	0	0	0	1
Tree holes	5	0.9524	2	1	0	0	0	0	0	0	0	0

Table S4. Summary of surveys of mammals during the dry and rainy seasons. Q1 to Q10 shows the first ten incidence frequency counts.

Water body	Observed species richness	Sample coverage	Q1	Q2	Q3	Q4	Q5	Q6	Q7	Q8	Q9	Q10
Dry season												
Waterholes	17	0.9969	2	1	0	0	1	2	2	0	0	0
Rock pools	16	0.9957	1	2	1	2	0	0	0	1	0	1
Tree holes	8	1	0	0	1	0	1	0	1	0	1	1
Rainy season												
Waterholes	16	0.9964	2	1	2	0	0	1	0	0	0	0
Rock pools	14	0.995	2	0	0	0	0	0	0	0	0	2
Tree holes	6	1	1	0	0	0	0	0	0	1	0	0

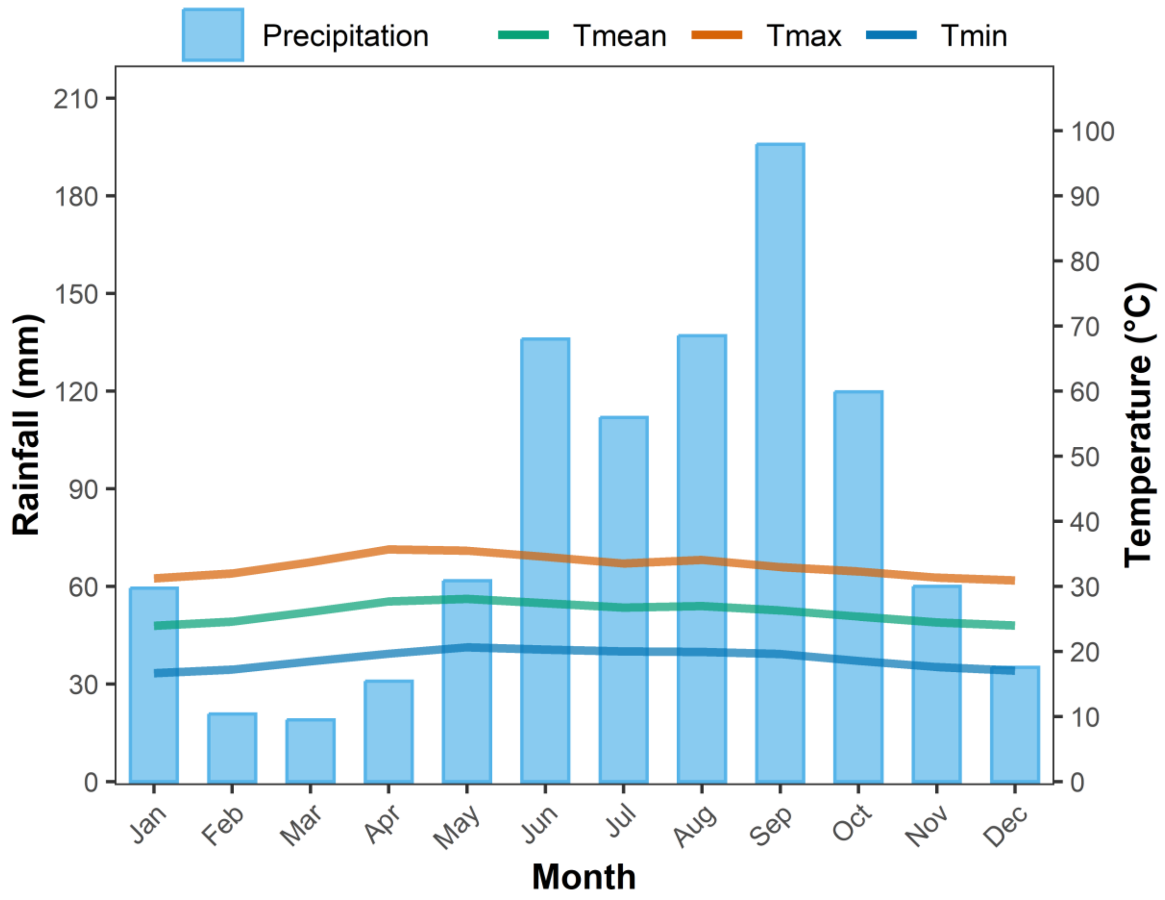

Figure S1. Historical average monthly precipitation and temperature in the Calakmul region (1979-2018).

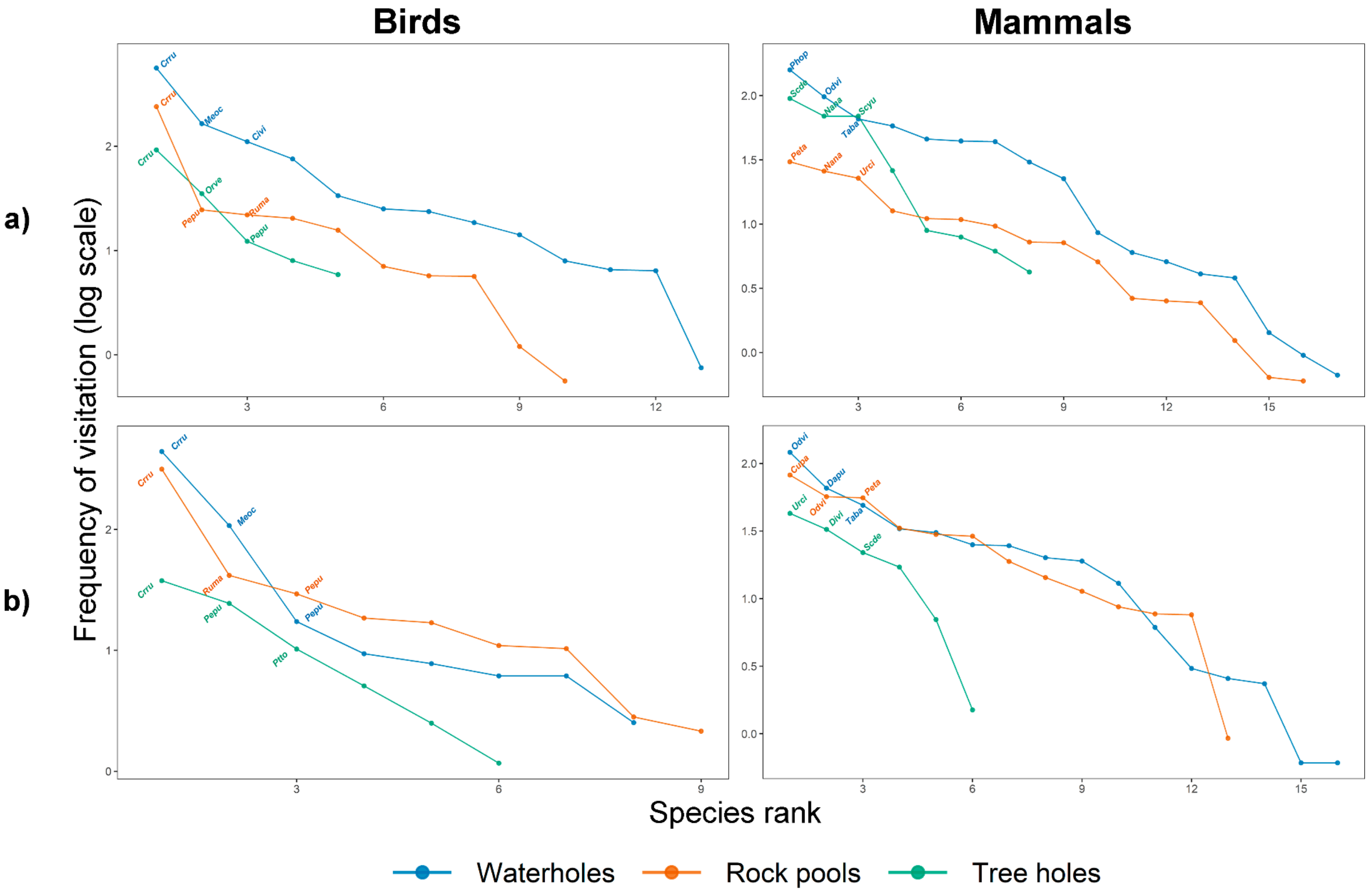

Figure S2. Rank-frequency curves of birds and mammals during the (a) dry and (b) rainy seasons in the different water bodies. Bird species codes: *Crru* = *Crax rubra*, *Civi* = *Ciccaba virgata*, *Meoc* = *Meleagris ocellata*, *Orve* = *Ortalis vetula*, *Pepu* = *Penelope purpurascens*, *Ptto* = *Pteroglossus torquatus*, *Ruma* = *Rupornis magnirostris*. Mammal species codes: *Cupa* = *Cuniculus paca, Dapu* = *Dasyprocta punctata, Divi* = *Didelphis virginiana, Nana* = *Nasua narica, Odvi* = *Odocoileus virginianus, Peta* = *Pecari tajacu, Phop* = *Philander opossum, Scde* = *Sciurus deppei, Scyu* = *Sciurus yucatanensis, Taba* = *Tapirus bairdii, Urci* = *Urocyon cinereoargenteus*.

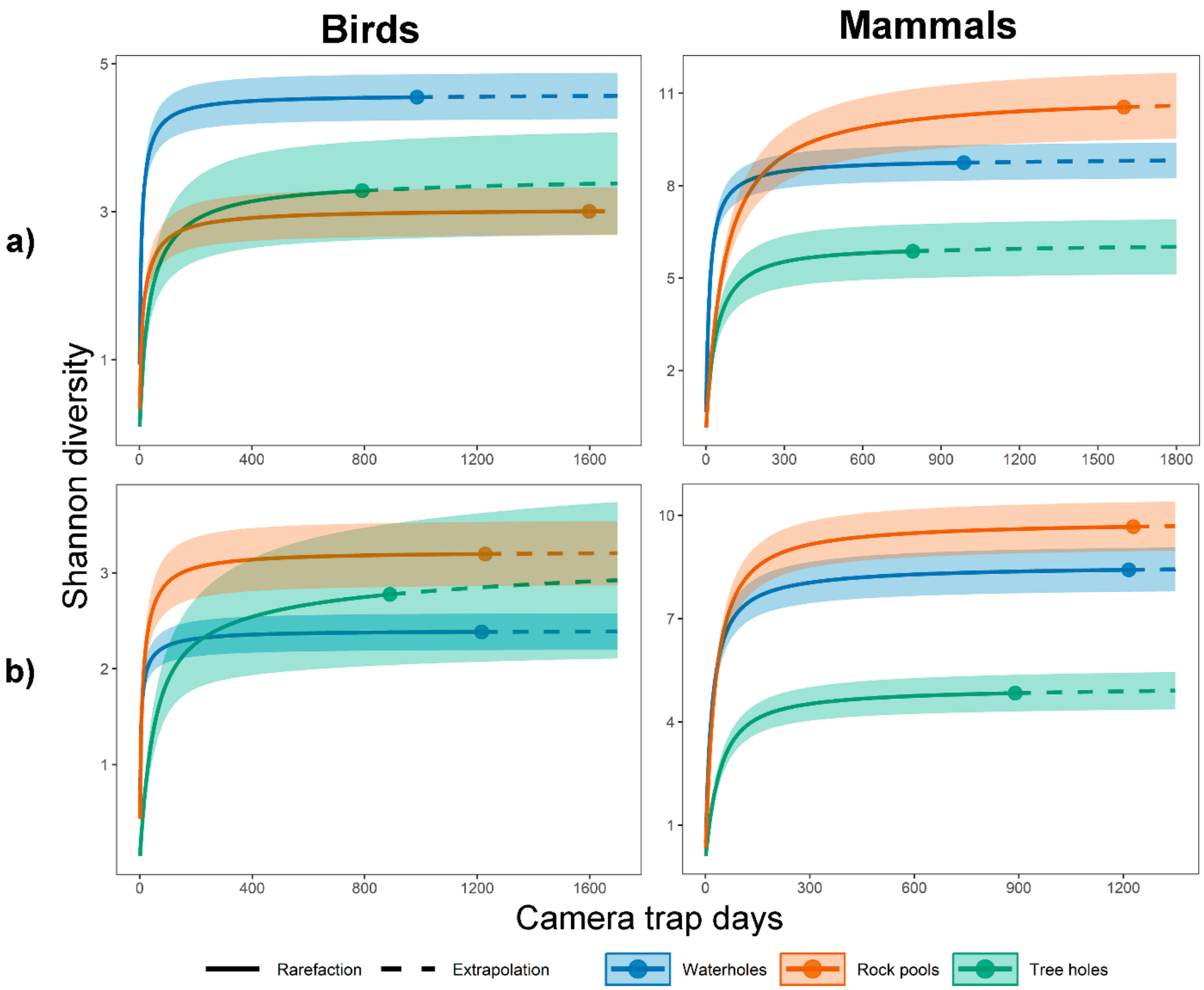

Figure S3. Comparison of Shannon diversity of birds and mammals that visited the water bodies during the (a) dry and (b) rainy seasons.

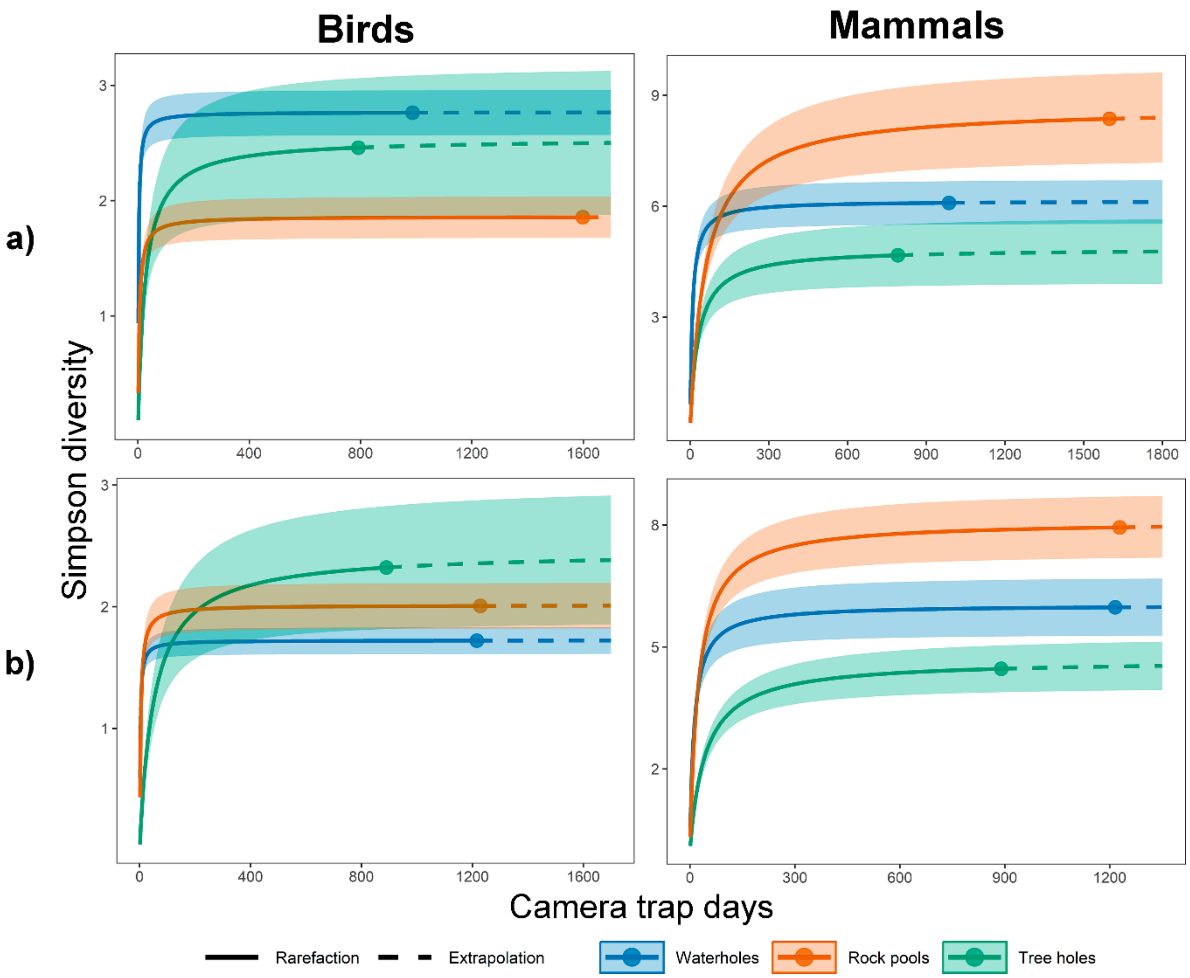

Figure S4. Comparison of Simpson diversity of birds and mammals that visited the water bodies during the (a) dry and (b) rainy seasons.

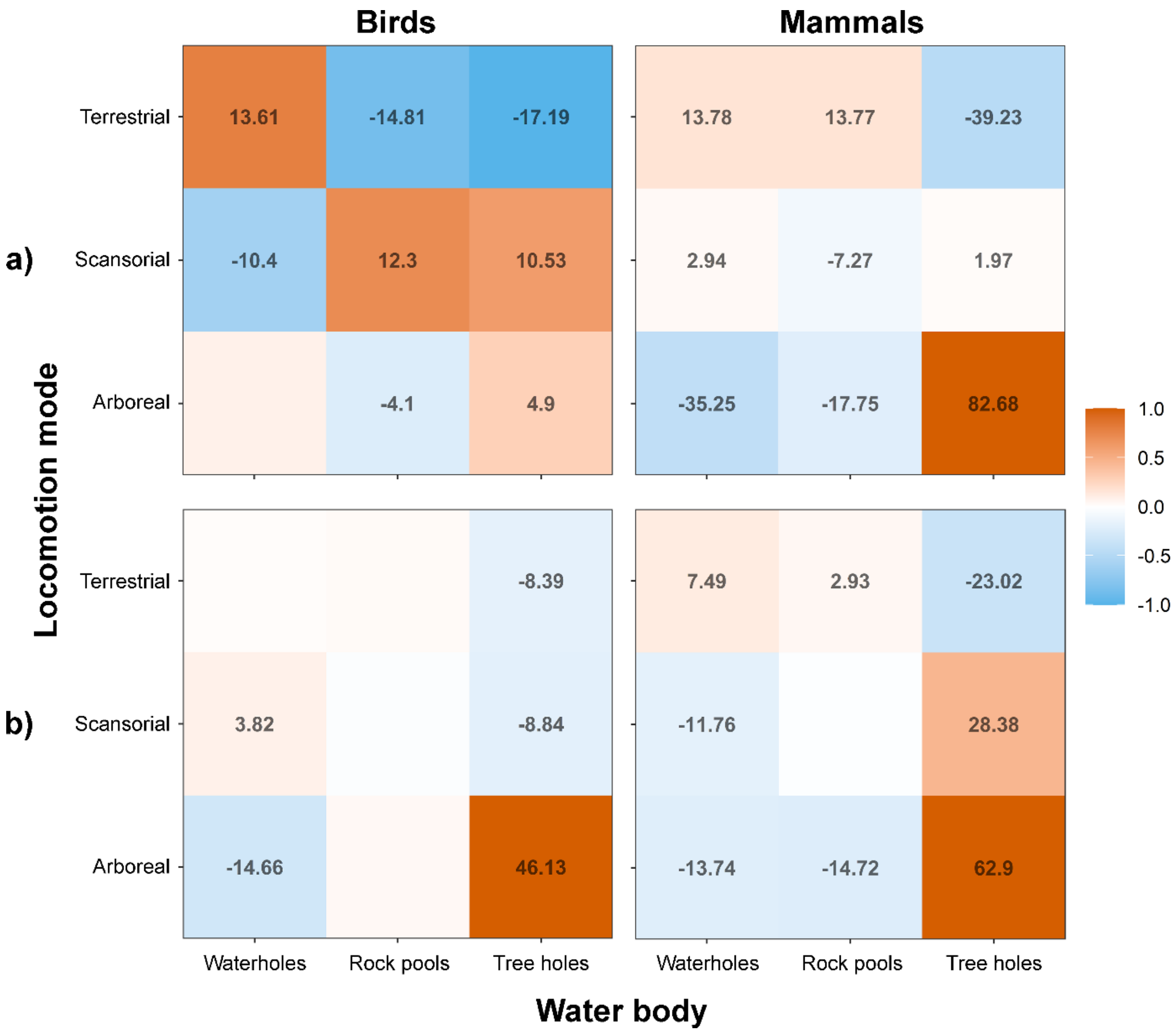

Figure S5. Standardized differences between observed and expected capture frequencies of birds and mammals by locomotion modes during the (a) dry and (b) rainy seasons. Only absolute values exceeding the critical value (1.96) are shown, indicating a significant difference at a significance level of $p < 0.05$. Positive or negative values indicate the direction of the differences.

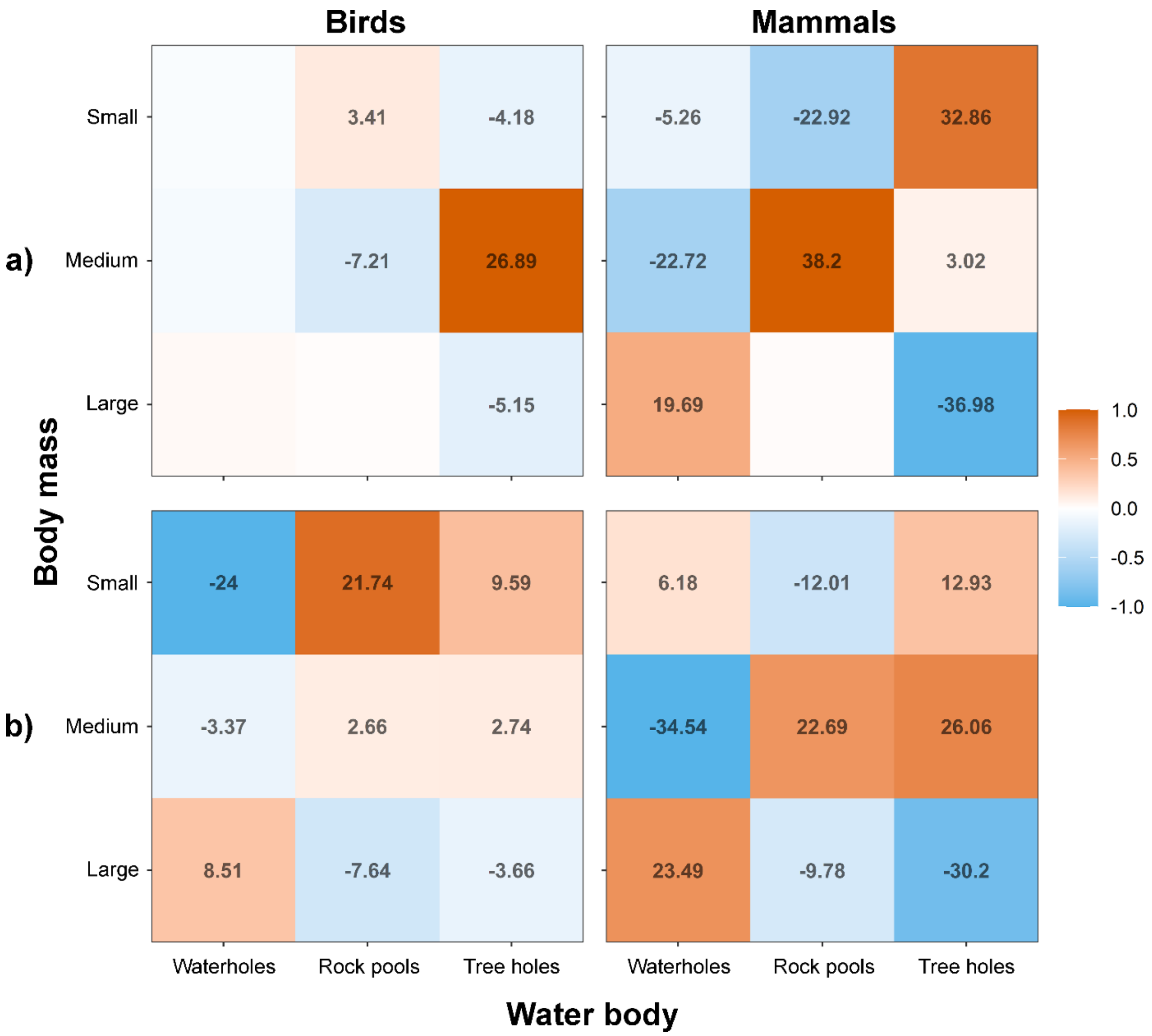

Figure S6. Standardized differences between observed and expected capture frequencies of birds and mammals by body mass categories during the (a) dry and (b) rainy seasons. Only absolute values exceeding the critical value (1.96) are shown, indicating a significant difference at a significance level of $p < 0.05$. Positive or negative values indicate the direction of the differences.
